# Retraction: *Tlr5* deficiency exacerbates lupus-like disease in the MRL/*lpr* mouse model

**DOI:** 10.3389/fimmu.2024.1455372

**Published:** 2024-07-03

**Authors:** Frontiers Editorial Office

Following publication, concerns were raised regarding the integrity of the data in the published figures and the validity of the data presented in the article.

The authors assisted in the investigation of this manuscript, which was conducted in accordance with Frontiers’ policies. Given the concerns about the validity of the data, the editors and the authors no longer have confidence in the findings presented in the article and concur with the retraction.

This retraction was approved by the Chief Executive Editor of Frontiers. The authors regret any inconvenience this may have caused to the reviewers, editors, and readers of Frontiers in Immunology.

